# Collection of fluorescence from an ion using trap-integrated photonics

**DOI:** 10.1038/s41377-025-02138-9

**Published:** 2026-01-29

**Authors:** Felix W. Knollmann, Sabrina M. Corsetti, Ethan R. Clements, Reuel Swint, Aaron D. Leu, May E. Kim, Patrick T. Callahan, Dave Kharas, Thomas Mahony, Cheryl Sorace-Agaskar, Robert McConnell, Colin D. Bruzewicz, Isaac L. Chuang, Jelena Notaros, John Chiaverini

**Affiliations:** 1 Massachusetts Institute of Technology, Cambridge, MA USA; 2 Lincoln Laboratory, Massachusetts Institute of Technology, Lexington, MA USA; 3 University of Oxford, Oxford, UK

**Keywords:** Single photons and quantum effects, Optical physics

## Abstract

Spontaneously emitted photons are entangled with the electronic and nuclear degrees of freedom of the emitting atom, so interference and measurement of these photons can entangle separate matter-based quantum systems as a resource for quantum information processing. Since confinement in a single-mode facilitates the photon interference needed for generating entanglement, the dipole emission patterns relevant in spontaneous emission present a mode-matching challenge. Current demonstrations rely on bulk photon-collection and manipulation optics that suffer from large component size and system-to-system variability—factors that impede scaling to the large numbers of entangled pairs needed for quantum information processing. To address these limitations, we demonstrate a collection method that enables passive phase stability, straightforward photonic manipulation, and intrinsic reproducibility. Specifically, we engineer a waveguide-integrated grating to couple photons emitted from a trapped ion into a single optical mode within a microfabricated ion-trap chip. Using the integrated collection optic, we characterize the collection efficiency, image the ion, and detect the ion’s quantum state. The integrated optic covers 2.18% of the solid angle and collects 1.97 ± 0.3% of the spontaneously emitted light incident on the grating for a total collection efficiency of 0.043% into a single-mode waveguide. This proof-of-principle demonstration lays the foundation for leveraging the inherent stability and reproducibility of integrated photonics to create, manipulate, and measure multipartite quantum states in arrays of quantum emitters.

## Abstract (web summary)

We collect fluorescence photons from a trapped ion into a chip-integrated single waveguide. We introduce a new grating design technique to extend the effective aperture of a large grating.

## Graphical abstract

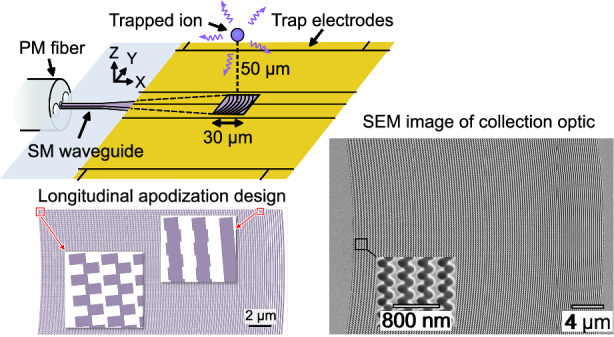

## Introduction

Individually confined atoms or ions form an effective platform for storage, processing, and measurement of quantum states due to their controllability and isolation from the environment^1^. Additionally, their abundance of optical transitions provides a mechanism to convert quantum information between matter and photonic states. This mechanism enables high-fidelity state preparation and measurement, as well as coherent, non-local connectivity. The detection of emitted single photons allows the projection of internal atomic states, but further manipulation of photon properties at the quantum level calls for single-mode guiding structures. The challenge of collecting isotropically emitted light into such single-mode structures limits the effectiveness of protocols generating entangled states for sensing, communication, or distributed quantum computing. Transferring emitted photons into single-mode optical fiber using bespoke, free-space lenses^2–4^ or high-finesse cavities^5,6^ has enabled proof-of-principle demonstrations of remote entanglement generation between trapped ions or atoms. However, the inherent challenges to robustness, phase and mode stability, and extensibility in bulk-optical approaches make them unlikely to allow high-fidelity creation of novel multipartite entangled states of many atomic systems^7^ or rapid coupling of large arrays of qubits in separate quantum information processing modules^8^.

Incorporating integrated optics may improve upon bulk optical approaches for the collection of spontaneous emission. Since ions can be trapped tens of microns above the chip surface in a microfabricated trap^9^, a grating integrated in that surface can achieve a numerical aperture similar to that of a free-space optic while minimizing lateral footprint because it is approximately 1000 times closer to the ion. The miniaturization of this approach makes it inherently scalable and well-suited for extensible quantum architectures. Each grating could collect light into a single-mode waveguide for integrated interference and photon-mediated entanglement. Alternatively, gratings could direct light to a waveguide-coupled detector placed far from the ion to allow readout via resonance fluorescence^10^ while mitigating adverse effects arising from the interplay of trapping and detector fields^11–13^. Furthermore, tailoring the spatial profile of collected light using multiple phase-stable integrated collecting optics can potentially mitigate sources of entanglement infidelity related to the finite temperature of confined atoms and ions^14,15^. Initial demonstrations using integrated photonic waveguides to *deliver* control light to trapped ions already highlight the benefits in path-length and beam-pointing stability when compared to traditional free-space optics^16–20^.

In this work, we collect ion fluorescence into a trap-integrated single-mode waveguide to demonstrate the key requirement for integrated photon-mediated entanglement. Because of the reciprocity of electromagnetic-wave propagation through passive linear structures without magneto optics, designing for light collection from a small volume can be re-framed as engineering a tightly focused emitted beam. To design a tightly focusing integrated optic, we introduce a novel method for tailoring the scattering strength of a diffraction grating to match a desired emission profile while also increasing the effective grating length and minimizing aperture-induced imperfections. We co-fabricate the grating with an ion trap and image its emission profile. By collecting photons emitted from the ion into a single-mode waveguide, we experimentally verify a model of grating collection efficiency based on ion position and emitted-photon polarization. As a proof of principle, we use the integrated collection path to detect 422 nm light from a ^88^Sr^+^ ion to determine its quantum state. This demonstration is an initial step toward high-performance remote entanglement generation and state detection of trapped ions with integrated collection optics. The demonstrated technique applies to all ion species of practical interest for quantum information processing and sensing, and could immediately be extended to trapped neutral atoms and other point source emitters. The following presents the grating design, characterization, and measurement, and then discusses the implications of our results.

## Results

**Fig. 1:**
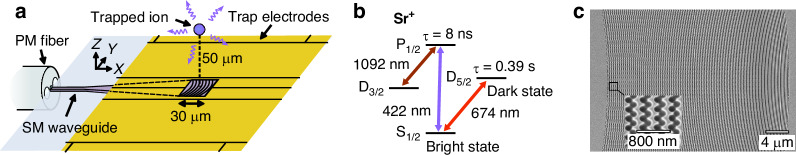
A focusing diffraction grating collects fluorescence photons from a trapped ion into a single-mode (SM) waveguide. **a** The schematic shows the grating’s placement beneath the trap electrodes and the path through a non-linear taper to convert from the 30 *μ*m grating width to a single-mode waveguide, which routes to an edge-coupled fiber. The ion height above the trap surface is 50 *μ*m (not to scale). **b** A simplified level structure shows the relevant transitions and excited state lifetimes. **c** The inset in this scanning-electron micrograph of the collection grating shows the detailed structure of phase-shift apodization

For our proof-of-principle demonstration (see Fig. 1), we fabricate ion traps with an integrated photon collection device using a 200 mm wafer-scale process that offers low losses at visible wavelengths^21^. The photonics platform consists of two layers of 100 nm-thick silicon nitride used for the grating and a layer of 100 nm-thick alumina used for low-loss photon routing; 90 nm of SiO_2_ separates each layer. The waveguide layers are clad above and below with 5 *μ*m-thick SiO_2_ layers. As shown in Fig. 1a, we deposit and etch a layer of aluminum above the upper cladding layer to define electrodes for RF and DC voltages that confine a single ^88^Sr^+^ ion 50 *μ*m above the chip surface (see simplified level structure in Fig. 1b). A 50-nm-thick transparent conductive film (indium-tin oxide) covers the dielectric (SiO_2_) exposed in gaps between metal electrodes above the grating. A quantizing magnetic field of 4 Gauss is applied in a direction normal to the trap surface.

### Grating device design

**Fig. 2:**
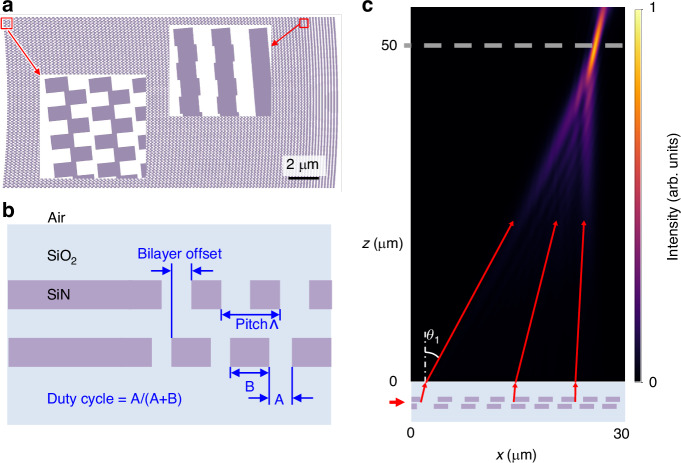
Dual-layer apodized diffraction grating design. **a** A focusing grating designed using phase-shift apodization. The insets show details of the phase-shift apodized grating teeth near the leading and trailing edge of the grating (see section S1.3 of the Supplement for details). **b** Cross-sectional structure of the dual-layer grating with materials indicated. Varying the grating pitch, *Λ*, provides longitudinal focusing (as shown in **c**). The bilayer offset breaks the vertical symmetry to obtain high upward directivity. **c** The vertical slice of the simulated grating emission shows longitudinal focusing to a small spot at the targeted ion height of 50 *μ*m

Designing and fabricating photonic devices to efficiently collect photons from an atomic point source at a working distance of tens of microns is challenging because it requires both low losses and sub-wavelength feature sizes at the short wavelengths relevant for ion transitions^21^. The collection efficiency can be mapped to the projection of the normalized electric field emitted by the optic onto the ion’s radiating-dipole emission pattern^14^. Assuming constant diffraction efficiency, minimizing the focused spot size at the ion height thus maximizes the collection efficiency. Therefore, we optimize the grating parameters visualized in Fig. 2 to create a tight focus by varying the grating pitch and setting the curvature of the grating teeth to concentrate rays both longitudinally and transversely^22,23^. Furthermore, maximizing upward directivity maximizes the fraction of light emitted toward the ion and prevents uncontrolled reflections from the lower interfaces in the chip structure that would not optimally couple to the ion. We achieve upward directivity by designing our gratings using two waveguide layers with a relative lateral shift in the propagation dimension (Fig. 2b) to break the vertical symmetry^23–26^. Finally, we tailor the grating amplitude profile to maximize the overlap with the ion emission incident on the grating, as depicted in Fig. 2a, c. We describe the full optimization sequence in the Supplementary Information.

Existing techniques for apodizing (tuning the local grating strength) to tailor the grating amplitude profile over a large grating size include varying the duty cycle of the etch within a single period or varying the depth of the etch^27,28^. However, gratings designed using these existing techniques can be difficult to fabricate, as they require feature sizes substantially below the optical wavelength, and most relevant electronic transitions in ions have short wavelengths. We have therefore developed a novel apodization method that enables continuous variation of the grating strength up to a maximum value without the need for feature sizes inaccessible to typical photolithography. Our technique subdivides the grating into sub-wavelength zones just under half a period in width (Fig. 2a). Shifting every other zone longitudinally by a continuously tunable distance shifts the phase of the locally emitted light such that scattering from neighboring zones partially or fully destructively interferes. We describe the detailed implementation in section S1.3 of the Supplementary Information. The only fundamental requirement is that the transverse period must be less than the wavelength of the light in the medium to prevent transverse diffraction. Using this technique, which we call phase-shift apodization, we design a grating with an emission profile that strongly suppresses extraneous longitudinal beam structure and closely matches the ion radiation mode (see Fig. 2c).

### Grating device characterization

**Fig. 3:**
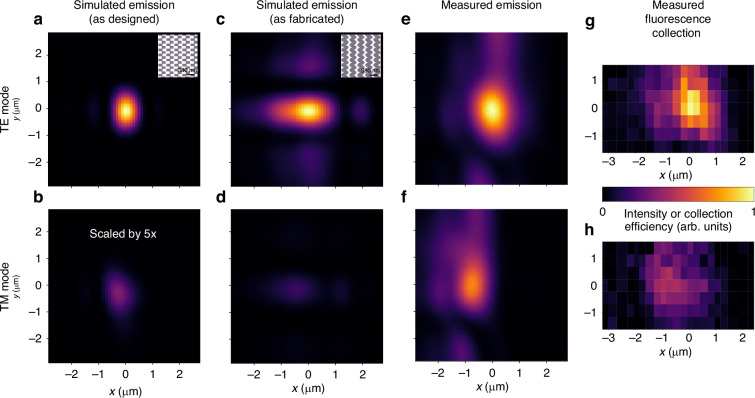
Spatial profiles of grating emission and collection. The columns depict the transverse-electric (TE) polarization mode (top row) and the transverse-magnetic (TM) polarization mode (bottom row) intensity profiles at the designed ion height (50 *μ*m) above the trap for the simulated emission of the ideal grating (**a**, **b**), the simulation of the grating taking into account known fabrication defects (**c**, **d**), the measured emission of the fabricated device (**e**, **f**), and the measured ion fluorescence collection of the fabricated device (**g**, **h**). The intensity scale in each column is normalized to the maximum of the TE emission/collection, which is stronger (as designed) for this device. Note that the ideal grating TM mode intensity (**b**) has been scaled up by 5 × to make it clearly visible since the peak intensity of the TM mode for the ideal grating is only 7% of the TE mode peak. The slight *y*-direction asymmetry visible in the simulated TM mode profile is due to an asymmetry about the *y* = 0 line in a subset of the subwavelength features in the phase-shift apodization pattern. In all cases, the TE mode is displaced relative to the TM mode, as expected. As described in detail in Methods section “Modeling fabrication effects in simulation”, the device simulated in (**c**, **d**) differs from the designed device in layer heights and grating tooth shape. The insets in (**a**, **c**) illustrate the deformation of the grating teeth from their original square shape. Finally, the device simulated in (**c**, **d**) includes an extraneous groove etched into the top SiO_2_ layer during processing of the wafer

The reciprocity of emission and collection allows us to characterize the expected fluorescence collection performance by profiling either the emitted or the collected mode (see Methods subsection “Theoretical description of ion fluorescence collection” for details on the calculation of the expected performance^8,14^). First, we verify the grating design using a 3D finite-difference time domain (FDTD) simulation of its emission profile, from which we calculate a 0.7% expected collection efficiency, which is a substantial fraction of the 2.18% solid-angle limit (Fig. 3a, b). Biases and unintentional alignment offsets in fabrication caused significant deviation from the designed geometry. We investigate the known discrepancies between the fabricated result and the ideal design using a second round of 3D FDTD and find an additional -8 dB loss, resulting in an expected collection efficiency of 0.11% (Fig. 3c, d). See Methods subsection “Modeling fabrication effects in simulation” for a detailed analysis. Next, we use an imaging system to profile the beam emitted by the fabricated device. Based on cross-sectional images of the beam at the target ion height, we calculate an expected collection efficiency of 0.041 ± 0.007% or −12.35 dB from the designed performance (Fig. 3e, f). The larger focused spot for TE emission of the fabricated device (FWHM in x: 1.15 *μ*m, FWHM in y: 1.66 *μ*m) relative to the design (0.58 *μ*m, 0.96 *μ*m) visually exemplifies the performance discrepancy (Fig. 3a, e).

Finally, we trap an ion over the collection grating and displace the ion to spatially map the fluorescence collection efficiency of the device (Fig. 3g, h). A single-mode fiber coupled to the waveguide at the chip edge routes the collected light to a photodetector (photo-multiplier tube, PMT). To determine the integrated detection efficiency we measure the efficiency of the traditional, free-space detection pathway using a high NA compound lens outside the vacuum system and multiply it by the ratio of photon counts measured on the integrated and free-space detection pathways. Using the measured detection efficiency and accounting for detector and transmission losses, we determine a single-mode collection efficiency of 0.043 ± 0.007% for the integrated collection grating. This value agrees with the calculation based on the emission profile within experimental uncertainty. See the Methods subsections “Grating characterization methods” and “Photon loss characterization” for a detailed accounting of the procedure and loss contributions.

We additionally characterize the transverse-electric (TE) and transverse-magnetic (TM) polarization overlap and the resulting crosstalk to determine the collection grating’s suitability for state detection and photon-mediated entanglement. For many atomic species, both polarizations are emitted equally in state detection; an optimally designed grating would thus maximize the collection efficiency for both TE and TM polarizations. On the other hand, photon-mediated entanglement often requires maximizing the collection from a desired ion transition, while suppressing collection from others. For the entanglement case, minimizing the overlap of TE and TM grating emission minimizes the incidence of potentially error-inducing crosstalk; additional photonic structures can further suppress unwanted TM crosstalk^8^. Our FDTD simulations show low crosstalk even though the maxima of the TE and TM modes are only 0.3 *μ*m (designed) and 0.5 *μ*m (as fabricated) apart since the grating emits much less of the TM mode (TM/TE ratio designed: 0.12; as fabricated: 0.18). We calculate a 13 dB suppression of TM crosstalk at the maximum of the TE emission for the designed grating and 9 dB for the simulation of the fabricated grating. We find that the ratio of power diffracted for the TM mode versus the TE mode is larger in measurements than in simulation (measured TM/TE ratio: 0.69). Therefore, our measurements of the device show greater crosstalk for both ion collection and emission. Here, we measure the TM crosstalk at the optimal location for TE to be −5.3 dB for both methods, while the offset between the maxima in *x* is 0.7 *μ*m for emission and 0.8 *μ*m for collection. The ratio discrepancy between simulation and measurement may be explained by potential deviations from the designed device that we do not include in our simulation, such as voids in the oxide above the grating teeth or over-etching of the grating teeth.

### State detection

**Fig. 4:**
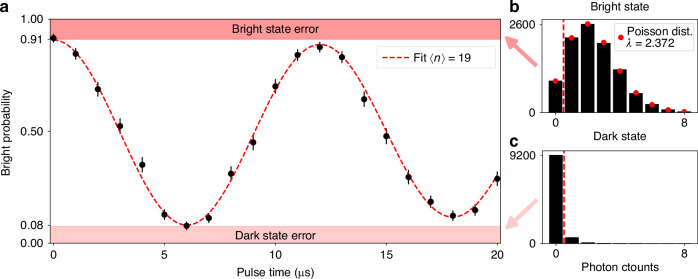
Rabi oscillations of a Sr^+^ optical qubit measured via fluorescence collected using a tailored diffractive grating coupler. **a** We use the integrated collection pathway to determine the ion’s state after excitation of the S_1/2_ to D_5/2_ transition at 674 nm. We plot the probability of measuring the optical qubit to be in the ground (bright) state as the length of the Rabi excitation pulse is scanned. The excited state is not resonant with the readout light, so an ion in this state remains dark. Thermal motion of the ion causes the decay of the Rabi oscillations. The dashed line shows a fit assuming an average excitation of the lowest-frequency motional mode 〈*n*〉 of 19 quanta, close to the Doppler-cooling limit. Panels (**b**, **c**) show state detection histograms for bright (91% fidelity) and dark (92.5% fidelity) states. We classify a result as bright if we detect at least one photon in the 8 ms detection time. The bright-state histogram is well-characterized by a Poisson distribution with mean *λ* = 2.372

As a proof of principle demonstration, we detect the state of a trapped ion by coupling the integrated collection device, via optical fiber, to an external photomultiplier tube. We distinguish between the $| 1\left.\right\rangle =| {\mathrm{S}}_{1/2},m=-1/2\left.\right\rangle$ (bright) and $| 0\left.\right\rangle =| {\mathrm{D}}_{5/2},m=-5/2\left.\right\rangle$ (dark) states by illuminating the ion with 422 nm light resonant with the S_1/2_ → P_1/2_ transition in Sr^+^ that scatters rapidly in the bright state and does not scatter in the metastable dark state (Fig. 1b). Figure 4b, c show the detection histogram for an ion initialized in the S_1/2_ level and for an ion shelved in the $| 0\left.\right\rangle$ state, respectively, for an 8 ms detection time. The bright state follows the expected Poisson distribution, while the dark state distribution is non-Poissonian because it includes trials where the ion spontaneously decayed back to the $| 1\left.\right\rangle$ state during the detection window (2%) and trials where shelving failed (0.5%). We use an 8 ms detection time with a one-photon threshold to balance the bright signal against errors caused by dark counts and decay of the metastable state (*τ* = 0.39 s lifetime). These parameters allow us to detect the dark state with 92.5 ± 0.3% and the bright state with 90.7 ± 0.3% fidelity. We can detect the bright state faster using an adaptive protocol that classifies a trial as bright after the first count^29^. Splitting the 8 ms detection time into 10 bins allows us to classify a bright state in an average of 2.66 ms and thus an average of 5.33 ms to detect an equal mixture of bright and dark states. To complete our demonstration, we use the integrated pathway to measure the ion’s state during excitation on the $| 1\left.\right\rangle \to | 0\left.\right\rangle$ transition after cooling close to the Doppler limit and preparing in the $| 1\left.\right\rangle$ state (Fig. 4a).

The achieved state detection duration and fidelity are limited by the observed detection efficiency. Of the −47.7 dB total photon loss, we attribute −16.6 dB to the solid angle subtended by the grating aperture, −17.1 dB to imperfect mode matching of the grating, −8.5 dB to transmission losses (dominated by ~5.5 dB loss at the waveguide to fiber interface), and −5.5 dB to the detector’s quantum efficiency (see Table 1 and Methods subsection “Photon loss characterization”). Of the −17.1 dB mode matching loss, −2.1 dB is expected based on the grating design, −0.3 dB comes from the addition of an ITO layer for electrical shielding, and the remaining −14.7 dB originate from imperfections and miscalibrations in the fabrication, such as the accidental introduction of a 500 nm deep divot in the top SiO_2_ and other effects explained in Methods subsection “Modeling fabrication effects in simulation”. We observe a signal-to-background ratio of 36 with a bright rate of 297 s^−1^ and a dark rate of 8.1 s^−1^. The background count rate consists of 4.3 s^−1^ from scatter of the excitation laser, 3.0 s^−1^ detector dark counts, and 0.8 s^−1^ other background. Lowering transmission and detector losses would proportionally increase the signal and background from laser scatter, but improving the definition of the grating would increase both the signal and signal-to-background ratio since a smaller fraction of laser scatter would couple into the waveguide.

**Table 1:** Summary of photon losses

	Ion measurement (dB)	Emission-based calculation (dB)	With known improvements (dB)
Count ratio (integrated to free-space)	−27.34 ± 0.04	-	-
Detection efficiency (free-space)	−20.34 ± 0.1	-	-
Loss from grating input to detector	-	−8.5 ± 0.7	−5
Detector quantum efficiency	-	−5.5 (PMT)	−1.6 (SPAD)
Collection calculated from emission profile	-	−33.9	−21.5
Total:	−47.68 ± 0.11	−47.9 ± 0.7	−28.1

We measure the ion fluorescence collection by calibrating the efficiency of the traditional free-space detection and multiplying the result by the ratio of counts on the integrated and traditional collection pathways. The ion measurement agrees well with the expected performance based on the grating’s emission profile and the routing and detection losses. See Methods subsection “Photon loss characterization” for a detailed explanation of the measurements and losses. The right-most column displays the potential performance of the same device with improved fabrication fidelity, the use of a single-photon avalanche diode (SPAD) photon detector instead of a photo-multiplier tube (PMT), and the use of a multimode fiber instead of a single-mode PM fiber for coupling between the chip and detector

## Discussion

In this work, we demonstrate the first collection of fluorescence from a trapped ion into a single-mode waveguide integrated into the trap chip. We introduce a grating design methodology that allows us to increase the effective solid-angle subtended by the collection grating and improve the grating’s longitudinal focusing, and thus the collection performance. We fully characterize the designed and fabricated device’s performance with numerical simulation, imaging of the grating emission, and profiling of ion fluorescence collection. This characterization allows us to experimentally validate the collection efficiency model presented in ref. ^14^. We model the effects of fabrication defects and experimental imperfections and understand their contributions to the detection efficiency (see Methods subsection “Modeling fabrication effects in simulation”). This understanding lays the foundation for dramatic improvement of the performance of future devices. We study the TE/TM mode overlap of both the design and the fabricated device, finding that the design suppresses crosstalk as desired for use in photon-mediated entanglement protocols^3,8^. Finally, we use the integrated collection pathway to measure the electronic state of an ion. The low background level in our measurement verifies the potential for this pathway to have the signal-to-noise ratio required for high-fidelity state detection.

The verification of our design and the validation of our collection models allows us to project performance with straightforward improvements to address current limitations of the device. Fabricating the grating with the designed layer heights and features would increase the collection efficiency by up to 12 dB (see Methods subsection “Modeling fabrication effects in simulation” for details). Using a single-photon avalanche diode with 70% quantum efficiency (QE) instead of a photo-multiplier tube (PMT) with 28% QE would increase the detection rate by 4 dB. Using a multimode fiber to couple photons from the waveguide to a detector could eliminate 3.5 dB or more of the current ~ 5.5 dB facet loss. These potential improvements to the current device could increase the photon detection efficiency by 19.5 dB without any design changes (see Table 1). Scaling the fluorescence count rates by the full 19.5 dB and non-detector background count rates only by the 7.5 dB from routing and detection improvement would result in a detection time under 350 *μ*s with over 0.999 fidelity. For photon-mediated entanglement of ions in different zones of a trap chip, the relevant number is the projected total detection efficiency of −28.1 dB (see Table 1). For typical atomic-physics parameters, and excitation rates of approximately 1 MHz, this detection efficiency would lead to a few coincidences per second^2,3,8^ without any further optimization of the grating or system design.

Future designs could further improve performance through increased grating size and higher-resolution lithography to create a tighter focus and thus more efficient mode matching. Gratings covering over 10% of the ion’s solid angle are compatible with standard ion trap architectures^8^. In this work, we have designed structures that achieve collection efficiencies above 60% into a single-mode structure in simulation. The integration of waveguide-coupled detectors could eliminate facet-coupling losses and achieve QE approaching unity^30^, which would imply a photon detection efficiency of approximately 5% for a grating covering 10% of the solid angle. Tiling multiple gratings in readout or entanglement-generation zones could further increase the solid angle subtended^8^. Furthermore, future designs may be tailored for either state determination or photon-mediated entanglement by exploiting the different requirements for polarization or laser-scatter to background discrimination. Together, these improvements have the potential to increase the probability of detecting an emitted photon close to the solid angle limit of a grating subtending a large area below the ion and thus increase the expected photon-mediated entanglement rates beyond the level shown in recent free-space demonstrations with photon detection efficiencies of approximately 2.5%^3,4^. Photonic integration would additionally enable more straightforward incorporation of spatial multiplexing via arrays of ion pairs using photon-collection waveguide optics. Such multiplexing could allow for a near-linear increase in entanglement rate as a function of array size. In summary, this proof-of-principle experiment demonstrates the feasibility of compact and modular fluorescence collection and coupling into integrated single-mode waveguides, with applications to state readout and photon-based entanglement in scalable trapped-ion quantum processors^31–34^.

## Materials and methods

### Grating characterization methods

As mentioned in the Results section, we use three methods to characterize the collection device: 3D finite-difference time domain (FDTD) simulation, imaging of the emitted mode using a high-NA microscope objective, and imaging by scanning the position of a trapped ion while detecting fluorescence. This section will explain these methods in detail.

**Fig. 5:**
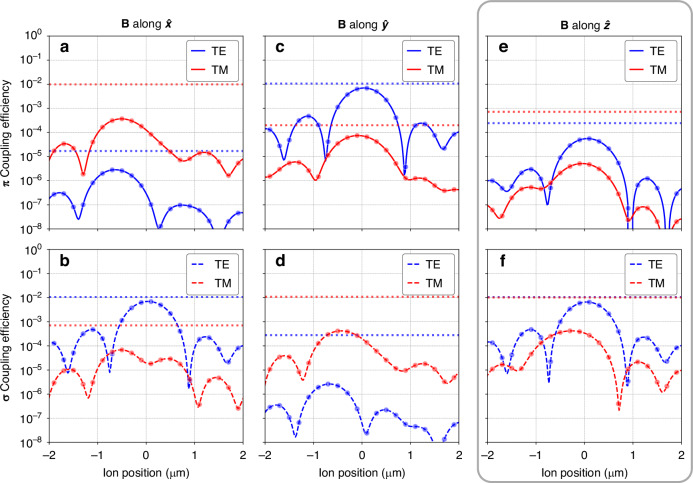
Simulations of ion emission coupling efficiency vs. ion displacement along the propagation axis. We investigate magnetic fields aligning the quantization axis along $\hat{x}$ (**a**, **b**), $\hat{y}$ (**c**, **d**), and $\hat{z}$ (**e**, **f**). Points are simulated data, and lines are interpolated splines to guide the eye. We highlight the $\hat{z}$-oriented magnetic field as that is the configuration used in our demonstration. The top row shows the collection of *π* emission as a fraction of the total ion fluorescence, and the bottom row shows the *σ* fraction. The peak coupling efficiency of ~ 0.67% for a given polarization approaches the solid-angle collection limit of ~ 1.09% for the fraction of ion radiation in a given polarization that lands within the 30 × 30 *μ*m^2^ footprint of the grating. Further design optimization could thus improve the coupling efficiency by no more than ~ 50%

To determine the expected performance of the collection grating, we simulate multiple grating geometries starting with the geometry dictated by the design process detailed in the previous section. For 3D FDTD grating simulations, we launch a single mode of either transverse electric (TE, top row of Fig. 3) or transverse magnetic (TM, bottom row of Fig. 3) polarization into the start of the 30 × 30 *μ*m^2^ grating— the performance of the non-linear taper is simulated separately. We then record the field emitted upwards by the grating just above the chip surface, since simulation of the entire air/vacuum volume up to the target ion height would require significant resources. We use far-field projection to generate the mode images at the nominal ion height (Figs. 2c and 3). We verify our calculation of the expected collection based on the normalized field profile (see Section "Theoretical description of ion fluorescence collection") by calculating the overlap of the simulated grating field with the ion radiation field at a location just above the chip surface and find good agreement between the two methods. We show the results of this overlap calculation for different choices of the quantization axis in Fig. 5.

Beyond the ideal-case simulation, we simulate a variety of grating and chip-surface geometries that are motivated by SEM images of the grating to account for fabrication-induced discrepancies in grating performance. In Fig. 3 we compare the performance between our ideal-grating simulation (as designed) and the simulation including the all of the fabrication effects we investigated (as fabricated). For the depicted “as fabricated” simulation, we accounted for the addition of a thin indium-tin oxide (ITO) film deposited on the surface of the ion-trap chip as a transparent conductor to shield the ion from exposed dielectric material, deviations in the thicknesses of the cladding and guiding layers from the design, and geometric deformations of the grating teeth. Further details and analyses are provided in the next section.

After fabrication, we measure the emission profile of the physical device. For this measurement, we send light into the grating from an edge-coupled polarization-maintaining (PM) fiber and image the emitted beam with a 50X, 0.95 NA microscope objective. By collecting images at a range of vertical offsets, we construct a 3D beam profile. To compare the TE and TM emission in relative intensity and position, we rotate the polarization coupled into the fiber by 90 degrees and re-measure the emitted beam without disturbing the imaging setup. Figure 3e, f show a cross-section of the emitted beam at the nominal ion height for TE and TM light.

Once we have characterized the emission of the device, we measure its ion-fluorescence collection efficiency. We package the trap chip using a V-groove array of PM fibers that edge-couple to waveguides on the trap, and we install it in a cryogenic vacuum chamber as described in ref. ^20^. The PM fiber routes the collected light to a photo-multiplier tube (PMT) outside of the vacuum chamber for detection. We control the ion with light delivered by free-space optics. We measure the integrated fluorescence collection by driving the ion on the 422 nm transition and include a repumping laser beam at 1092 nm to make it a closed, cycling transition. We detect the emitted 422 nm photons using both the integrated pathway and our traditional imaging setup with a high-NA objective external to the vacuum system. By varying the DC electric potential, we raster the position of the ion in a plane at *z* = 50 *μ*m to spatially map out the collection in *x* and *y*. To distinguish the TE and TM components of the collected light, we place a polarizer before the PMT and repeat the collection map while accepting polarizations corresponding to either TE or TM (Fig. 3g, h).

### Modeling fabrication effects in simulation

As discussed in the Results section, the collection efficiency of the fabricated grating is approximately 12 dB lower than the designed efficiency. We therefore anticipate that we can substantially improve the efficiency by more faithfully reproducing the design in fabricated devices. To this end, we aim to quantify the impact of known deviations between our designed and fabricated chip geometries and, in turn, quantify the improvements we can expect from correcting for these deviations. We begin by simulating the ideal grating and chip geometry and sequentially introduce known deviations, re-simulating the cumulative effect each time to extract the effect of each deviation. We use the collection efficiency into the TE mode of the grating as our figure of merit, as it is the dominant collection contribution and obviates the need to run simulations for both TE and TM modes. For the designed geometry, we calculate a 0.67% (−21.75 dB) TE collection efficiency (Fig. 5f).

First, we consider the surface of our trap chips. A 50-nm film of indium-tin oxide (ITO, a transparent conductor) covers the chip surface to shield the ion from the exposed SiO_2_, with a gap through the center to prevent shorting of the trap chip’s electrodes. We shift the ITO in the *y* dimension of the grating by 1 *μ*m, to account for a measured offset between the chip’s surface-metal and waveguiding layers. For this geometry, our simulated collection efficiency is −22.05 dB (Δ_ITO_ = −0.3 dB). This loss could be mitigated by reducing the ITO thickness. Next, we add a 500 nm-deep divot in the oxide within the ITO gap to account for a known divot introduced during fabrication of the chip’s surface electrodes, resulting in a −24.95 dB simulated collection efficiency (Δ_divot_ = −2.9 dB). This divot was introduced during reworking of the electrodes during the fabrication process and can be avoided in the future.

In addition to these surface effects, we incorporate deformations of the grating layers into our simulations. Based on SEM images of several chips both during and after fabrication, we observe that the upper silicon nitride layer thickness is typically reduced by 15 nm from design, the lower silicon nitride layer thickness is typically reduced by 20 nm from design, and the SiO_2_ gap between the two layers is typically increased by 35 nm from design. Furthermore, we observe deformations of the phase-shift apodized grating-teeth that we assign to two categories: triangular and ellipsoidal (see Fig. 1c). In the triangular-deformation case, the SiO_2_-filled gaps defining the grating’s teeth are under-etched during fabrication and turn out smaller and more triangular than designed. In the ellipsoidal-deformation case, these gaps are over-etched during fabrication and turn out larger and more elliptical than designed. We simulate both the triangular and ellipsoidal cases while also adjusting the layer and gap heights as discussed above. For the triangular case, the simulated collection efficiency is −27.25 dB (Δ_tri_ = −2.3 dB). We use the ellipsoidal case, with a simulated efficiency of −29.65 dB (Δ_el_ = −4.7 dB), for the grating in Fig. 3e, f because its simulated mode profile better matches the measured profile. For this grating, 63% (−2 dB) of the input light is diffracted upward. We use this value to estimate the efficiency of the fabricated grating as discussed in the Results and in Section “Photon loss characterization”.

In sum (Δ_ITO_ + Δ_divot_ + Δ_el_), we can account for 7.9 dB of the approximately 12 dB performance difference between the designed and fabricated grating by incorporating known fabrication effects into our simulations. As a result, we anticipate that mitigating these fabrication effects would improve the performance of future collection gratings. We expect that the 4 dB discrepancy arises from effects that we were not able to characterize and are thus not yet incorporated into our simulations. Potential effects include uneven surface topography of the ITO which could help explain the transverse de-focusing, non-uniformity in the tooth deformations, as well as potential air gaps in the grating layers.

### Theoretical description of ion fluorescence collection

An atomic ion with zero nuclear spin and a single valence electron that is excited to the P_1/2_ state has a 1/3 probability of decaying without changing the projection of angular momentum (*π* emission) and a 2/3 probability of decaying with a Δ*m* = ± 1 change in the angular momentum projection (*σ*^±^ emission). The combination of these two emission components is spherically symmetric in the case of unbiased excitation to the upper state (zero atomic polarization). At any angle, the emitted light can be decomposed into equal contributions of two orthogonal linear polarizations with no radial component. However, the distribution of *π*- and *σ*-photons is inhomogeneous and follows the dipole emission pattern ${\bf{E}}\propto \,{\mathrm{sin}}\,(\theta )\hat{{\mathbf{\theta }}}$ for *π*, while *σ* emission is distributed as ${\bf{E}}\propto ({\cos}\,(\theta)\hat{{\mathbf{\theta }}}\pm i\hat{\phi })$, where *θ* is the polar angle and *ϕ* the azimuthal angle from the quantization axis set by an external magnetic field.

When calculating the total collection of ion fluorescence into the grating modes, the solid angle subtended by the grating gives a firm upper bound. Along the *x* axis, the ion is located 28 *μ*m from the start of the 30 × 30 *μ*m^2^ grating. A thickness of 50 *μ*m of vacuum and 5 *μ*m of SiO_2_ cladding separates the ion from the grating in the *z* axis. The grating thus covers 2.18% of the solid angle. A diffraction grating coupled to a single-mode waveguide can collect into either the quasi-TE or the quasi-TM mode of the waveguide. A maximum of 1.09% could couple into each mode. For photon-mediated entanglement applications the magnetic field direction should be chosen to separate *π* and *σ* emission by maximizing the coupling into either the TE or TM mode and minimizing crosstalk from the other emission pattern into that mode. This strategy preserves the entanglement of an emitted photon coupled with the atomic state. Our demonstration uses a magnetic field oriented in the *z* direction, which results in predominantly *σ* emission (95.6%) incident on the grating with near equal contributions to the TE and TM polarization directions (see Fig. 5e, f).

The above description calculates the geometric limits on collection by a planar optic. To calculate the expected coupling efficiency (*η*) due to the mode overlap we use the formalism developed in^14^:

1 $$\eta =\frac{1}{16}{\omega }_{0}^{2}| {\bf{p}}\cdot {{\bf{E}}}_{g}^{* }({{\bf{r}}}_{0}){| }^{2}$$

where *ω*_0_ is the photon frequency, **p** is the dipole polarization vector and ${{\bf{E}}}_{g}^{* }({{\bf{r}}}_{0})$ is the electric field input into the grating normalized to unit power and evaluated at the position of the ion. The coupling is maximized by maximizing the projection of the normalized field onto the radiating dipole $| {\bf{p}}\cdot {{\bf{E}}}_{g}^{* }({{\bf{r}}}_{0})|$.

To calculate the expected coupling given a measured beam intensity profile we scale the polarization vector to emit unit power (${p}_{0}=\sqrt{\frac{3}{4{\pi }^{3}}\frac{{\lambda }^{4}}{{c}^{3}{\mu }_{0}}}$) [$\frac{Ams}{\sqrt{W}}$]^14^ and express the grating electric field amplitude normalized to unit power *E*_*g*_ [$\frac{V}{m\sqrt{W}}$] in terms of the intensity per unit area normalized to unit power *I*_*g*_ [$\frac{1}{{m}^{2}}$]:

2 $${E}_{g}=\sqrt{2c{\mu }_{0}}\sqrt{{I}_{g}}$$

Here *μ*_0_ is the vacuum permeability. In our case of predominant *σ* emission incident on the grating, we can approximate the dot product $| \hat{{\bf{p}}}\cdot {\hat{{\bf{E}}}}_{g}|$ as 2/3 from the Clebsch-Gordan coefficient from *σ* emission times 1/2 for the coupling to either the quasi-TE or quasi-TM mode. If the TE and TM intensity profiles are normalized by their respective total intensities and combined, then the maximum collection efficiency of Eq. (1) simplifies to

3 $$\eta =\frac{1}{4\pi }{\lambda }^{2}\frac{{\mathrm{I}}_{max}}{{s}^{2}}$$

defined by a maximum normalized intensity at the brightest pixel of I_*max*_ [unit-less] for a pixel of length *s*. We use this formula to calculate the expected coupling based on the simulated and measured beam profiles. We cross-checked Eq. (3) with the full 3D FDTD simulation of the overlap between the ion emission and the grating mode and found a <2% discrepancy, which we attribute to the assumption that all of the incident light is *σ* polarized.

### Photon loss characterization

To characterize the loss mechanisms in the integrated collection pathway, we first measure the overall detection efficiency by deterministically emitting single photons from the ion. Then we determine the different loss mechanisms which can be divided into the overlap of the photon with the grating mode, the losses incurred routing the photon from the grating to the detector, and the detector quantum efficiency of 28% (or −5.5 dB) taken from the PMT manufacturer specification for 422 nm light.

Due to the low efficiency, directly measuring the detection efficiency on the integrated pathway would be prohibitively slow, so we calibrate the traditional bulk-optics imaging system using deterministically emitted single photons from the ion and use that calibration to determine the integrated efficiency by the ratio of photon counts from the two pathways. To calibrate, we repeat the following procedure. Resonant 422 nm light pumps the ion into the metastable D_3/2_ state. A pulse of 1092-nm light clears the D_3/2_ state via the short-lived P_1/2_ state such that the ion deterministically emits a single 422-nm photon. During the time window of the clearing pulse, we monitor the photo-detector for the emitted photon collected via an F/1.1 compound lens outside the vacuum chamber window. We determine the background counts by immediately repeating the 1092 nm pulse and detection step. Dividing the background-subtracted photon counts by the number of trials returns the detection efficiency of the bulk-optics imaging system. To determine the detection efficiency of the integrated path, we illuminate the ion with both 422- and 1092 nm light and measure the counts detected using both the traditional and integrated paths. Multiplying the known detection efficiency of the traditional imaging system ((9.24 ± 0.22) × 10^−3^ or −20.34 dB) by the ratio of counts between the two systems ((1.85 ± 0.02) × 10^−^^3^ or −27.34 dB) returns the detection efficiency of the integrated path ((1.71 ± 0.04) × 10^−5^ or −47.68 dB) (Table 1). We also use this ratio method to measure the grating collection modes plotted in Fig. 3g, h to account for spatial variation in the observed ion fluorescence intensity.

The overlap of an emitted photon with the grating mode depends both on the fraction of the solid angle subtended by the grating and the mode-matching of the ion emission to the grating mode. We measure the combination of the two factors by imaging the beam emitted by the grating at the target ion height for both TE and TM input (Section “Grating characterization methods”). Since the ion emits equally into both polarizations, we combine the two normalized intensity profiles and use the method described in the previous section to determine the expected collection fraction of 0.041% or −33.9 dB. This value includes 2 dB loss to account for light that starts in the grating mode but is not diffracted upward (see Section “Modeling fabrication effects in simulation”). This subtraction allows more accurate comparison to the simulations of grating collection which include the loss from incomplete upward diffraction (see Table 1).

We determine the routing losses by back propagating light from the detector end and measuring the power in the grating emission. Starting at the detector end, the losses are comprised of transmission and mating sleeve loss in the PM fiber (~1 dB), reflections, scatter, and mode-matching loss at the waveguide-to-fiber edge coupler(~5.5 dB), transmission and bend losses in the waveguides (~1 dB), light lost in the layer transitions from silicon nitride to alumina waveguides (<0.6 dB), imperfect mode-matching in the non-linear taper to the wide grating mode (<0.5 dB), and, finally, incomplete upward diffraction (~2 dB, see above). These approximate values are derived from separate measurements and simulations. We used optical adhesive as an index-matching material to reduce the facet losses by 3 dB from 8.5 dB to the 5.5 dB we quote. The total loss we measure by back-propagating light is 10.5 ± 0.7 dB, which we subtract 2 dB from when quoting the routing loss in Table 1 to account for the incomplete upward diffraction that we already include in the grating collection efficiency (see paragraph above). We determine the total loss at the cryogenic operating temperature using our EMCCD camera to image the grating emission. We calibrate the EMCCD camera image by first measuring the emission at room temperature and comparing it with the result from a power meter. The dominant uncertainty in the loss measurement is the background on the power meter measurement caused by scatter at the fiber-waveguide interface.

There are three clear areas to greatly improve the efficiency of the collection and detection device without requiring a design change. Improving the fabrication to match the designed grating would reduce the mode-matching losses by about 12 dB (Fig. 3 and Table 1). Using a multimode fiber instead of PM fiber would greatly reduce the mode-matching losses at the waveguide-fiber interface. In similar systems we have seen the loss at this interface go down to 2 dB without index-matching material, which would be a ~3.5 dB improvement. Improving the facet surface and using index-matching material could further lower the interface loss below 2 dB by reducing scatter and reflections. Finally, replacing the PMT (28% quantum efficiency) with a single-photon avalanche diode (70% quantum efficiency) would reduce the losses by an additional 4 dB. We did not use a single-photon avalanche diode in our demonstration because the PMT had 10X lower dark counts and thus offered higher fidelity for state detection.

## Supplementary information

Supplementary information for collection of fluorescence from an ion using trap-integrated photonics

## Data Availability

Data used in the preparation of this manuscript will be made available upon request.
