# Supplementary material for: Collection of fluorescence from an ion using trap-integrated photonics
Source: Light Sci Appl. 2026 Jan 29;15:95. doi: 10.1038/s41377-025-02138-9 (PMC12855832; doi:10.1038/s41377-025-02138-9)
Supplement: Supplementary file 1 — Supplementary information for collection of fluorescence from an ion using trap-integrated photonics [file 41377_2025_2138_MOESM1_ESM.pdf]

Supplementary information for collection of fluorescence from an ion using trap-integrated photonics

Felix W. Knollmann,[1,*] Sabrina M. Corsetti,[1] Ethan R. Clements,[1] Reuel Swint,[2] Aaron D. Leu,[1,3] May E. Kim,[2] Patrick T. Callahan,[2] Dave Kharas,[2] Thomas Mahony,[2] Cheryl Sorace-Agaskar,[2] Robert McConnell,[2] Colin D. Bruzewicz,[2] Isaac L. Chuang,[1] Jelena Notaros,[1] and John Chiaverini[1,2]

[1] *Massachusetts Institute of Technology, Cambridge, MA 02139, USA*
[2] *Lincoln Laboratory, Massachusetts Institute of Technology, Lexington, MA 02421, USA*
[3] *University of Oxford, Oxford OX1 3PU, United Kingdom*

[*]*fwk@mit.edu*

S1. Design of a diffraction grating for integrated fluorescence collection

Passive photonic systems are reciprocal. A diffraction grating designed to maximize the light emitted towards an ion will thus maximally collect light emitted by the ion [8, 14]. This allows us to interchangeably design and characterize the grating performance as either a focusing light emitter or, reciprocally, as a light collector. We design the focusing grating used in this demonstration by first identifying optimal grating geometries for fixed diffraction angles, using these optima to design a focusing solution in 2D, and then expanding this design in 3D. In this section, we provide a detailed description of an apodization technique, phase-shift apodization, that we introduce to vary the grating's diffraction strength and match the ion's emission intensity profile over the length of the grating.

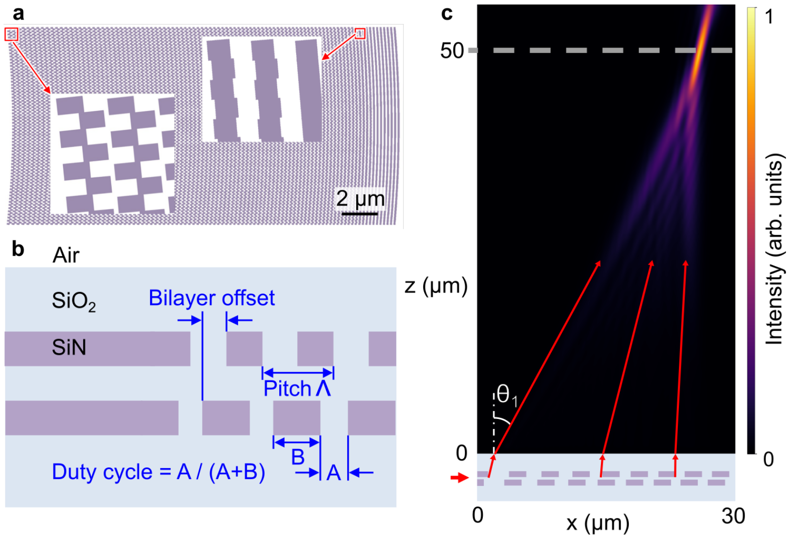

Fig. 6. Dual-layer apodized diffraction grating design. Figure reproduced from main text. **a** A focusing grating designed using phase-shift apodization. The insets show details of the phase-shift apodized grating teeth near the leading and trailing edge of the grating (see Methods subsection S1.2 for details). **b** Cross-sectional structure of the dual-layer grating with materials indicated. Varying the grating pitch, Λ, provides longitudinal focusing (as shown in panel **c**). The bilayer offset breaks the vertical symmetry to obtain high upward directivity. **c** The vertical slice of the simulated grating emission shows longitudinal focusing to a small spot at the targeted ion height of 50 μm.

S1.1. Diffraction grating parameterization and optimization

To maximize the intensity of the emitted beam at the ion location, we design the grating to emit with a unidirectional, focused beam profile. Whereas a single-layer grating will radiate symmetrically, above and below the plane of the waveguide, the illustrated bilayer grating (Fig. 6b) can be designed to preferentially radiate in one direction. In specific geometric cases, the diffraction in the downwards direction can be completely eliminated (e.g., offsetting the two layers of the grating by $\frac{\lambda}{4}$ in both x and z, where λ is the optical wavelength in the grating material). Complete suppression of diffraction in the undesired direction (down) is not possible for all combinations of wavelength and emission angle when the vertical bilayer separation is not a freely adjustable parameter, but directionality can be generally improved in any bilayer waveguide structure through optimal selection of the bilayer offset dx and the duty cycle in the upper (DCU) and lower (DCL) layers, as defined in Fig. 6b [26].

In addition to emitting unidirectionally, we desire the grating to focus at the ion location. To build a focusing grating, we first identify the range of angles needed to enable focusing in the propagation dimension (x) of the grating. Then, we construct a library of optimized geometric parameters (bilayer x offset dx, DCU, DCL) for upward emission at each of the angles within the required range. To find these optimal parameters, we iteratively simulate a short length of a fixed-period grating in 2D FDTD and use a particle-swarm algorithm to optimize the geometric parameters. The required 2D FDTD simulation volume is small, so typical simulation times are only a few seconds.

The particle-swarm algorithm maximizes the grating efficiency figure of merit $\frac{\kappa}{\alpha+\kappa}$, with grating coupling coefficient into the desired upward mode κ and excess loss to undesired diffraction orders α. We derive κ and α from P_T, the total power lost from the grating waveguide over length L, and P_D, the power diffracted into the desired diffraction order. We implemented constraints that exclude duty cycle solutions resulting in feature

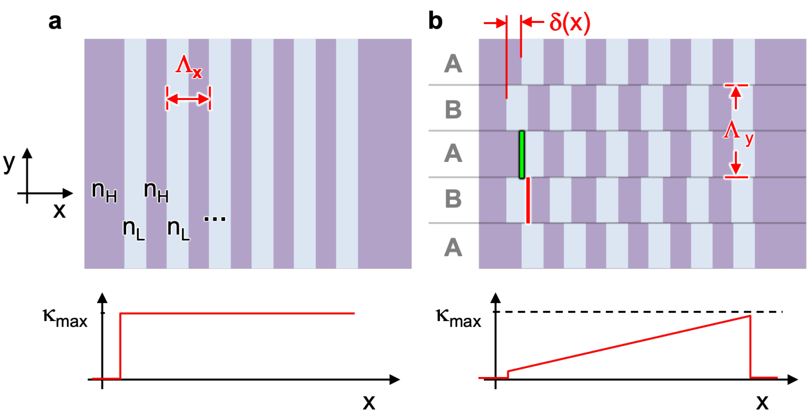

Fig. 7. Schematic illustration of conventional, **a**, and phase-shift apodized, **b**, gratings. The grating coupler is comprised of materials with refractive indices n_H and n_L and period Λ_x. The lower panels show the grating strength κ as a function of position within the grating. The phase-shift apodized grating coupler has been subdivided into zones A and B with a sub-wavelength period Λ_y. In a dielectric grating the leading edge (red) and trailing edge (green) of a grating line can produce scattering of equal magnitude but opposite phase. By shifting the grating features in zone B to be out of phase with those of A, net diffraction is reduced to zero since the combined scattering from two zones cancels out. Varying the phase shift thus continuously modifies the diffraction strength between zero and the maximum afforded by the materials' index contrast.

sizes less than the minimum critical dimension attainable in our fabrication process (0.12 μm). Thus, as the pitch approaches 0.24 μm, this feature size constraint forces the duty cycle of both layers to 0.5, and only the bilayer x offset dx is truly optimized. However, generally the average duty cycle for the two layers is near 0.5.

S1.2. Modifying diffraction grating strength: phase-shift apodization

The above simulations maximize the upward grating diffraction strength at a desired emission angle, and construct a suitably chirped grating that produces a focus, as illustrated in Fig. 6c. However, maximizing the coupling efficiency also requires tailoring the intensity distribution of the diffracted light by varying the diffraction strength along the length of the grating. In practice, this requires a mechanism known as apodization to locally reduce the coupling strength from its maximum value. Existing techniques for apodization include partial etching of the grating to various depths, adjustments to the duty cycle, and lowering the effective-index contrast via sub-wavelength patterning. The small features or varying etch depths used in these approaches typically require non-standard fabrication techniques [27, 28].

Here we describe a new apodization technique based on phase shifts (Fig. 7), where the grating is subdivided in the transverse (y) axis into two repeated zones (types A and B). This apodization technique is similar to that described in [27, 31, 32] for narrow, single-mode waveguides. We adapt it to wide, large-area waveguides. The resulting shape of the apodized pattern is similar to the sub-wavelength patterns described in [33] used for dispersion engineering. Shifting the features in one zone along the propagation axis by an amount δ changes the interference with light scattered from neighboring zones. This interference effect results in a tunable reduction of the diffraction strength by shifting between completely constructive and destructive interference. The only fundamental requirement is that the period of the zones in the y dimension Λ_y must be sub-wavelength ($\Lambda_y < \lambda_\mathrm{m}$) to prevent unintended diffraction in the yz plane. Note that the grating's effective index shortens the optical wavelength in the grating waveguide $\lambda_\mathrm{m} = \frac{\lambda_0}{n_\mathrm{m}}$, where λ_0 is the vacuum wavelength. Using this apodization technique, we can continuously vary the diffraction strength from zero to the maximum afforded by the refractive index contrast of the waveguide materials with feature sizes of just under $\lambda_\mathrm{m}/2$ in a single binary etch pattern. Although the phase-shift grating structure is intrinsically 3D, we model it in 2D using an effective index structure and repeat the optimization described in Section S1.1 for several discrete values of phase shift to create a library of optimal grating parameters for a 2D focusing grating.

S1.3. Longitudinal grating design

For optimal coupling, we tailor the diffraction strength along the length of the grating $\kappa(x)$ to produce a diffracted intensity profile that replicates the intensity profile $I_\mathrm{ion}(x)$ that would be produced in the inverse case of the ion ra-

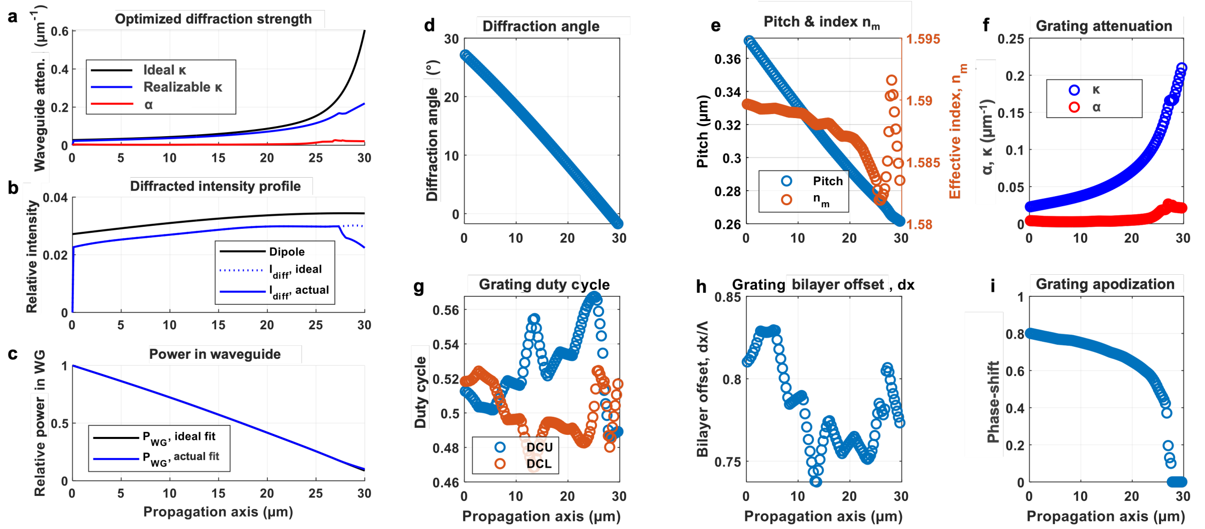

Fig. 8. Optimized 2D-grating parameters for coupling to an ion trapped 50 μm above the surface of a waveguide. **a** The library-constrained (red & blue) grating loss coefficients closely match the ideal (black) for most of the grating and approximate the ion dipole intensity profile for $x_{\mathrm{ion}} = 28$ μm (panel **b**). Panel **c** shows the success matching the ideal residual power propagating in the grating waveguide. The continuous solution of (**a**-**c**) is discretized to define a grating that varies from one cycle to the next to realize the desired diffraction angle **d**, requiring discrete pitches (**e**), and loss coefficients (**f**). The large ratio between α and κ demonstrates the efficiency of the grating design. The optimized grating duty cycle and bilayer offset are given in (**g**-**h**) and contain jumps resulting from the particle swarm optimization used. The phase-shift to realize the variation in κ as shown in (**a**, **f**) is given in **i**.

diating light on the grating surface. We can approximate the intensity profile diffracted from the grating waveguide along the propagation axis $I_{\mathrm{diffraction}}(x)$ as a continuous function of $\kappa(x)$ and $\alpha(x)$:

$$I_{\mathrm{diffraction}} = \kappa(x) \cdot e^{-[\kappa(x)+\alpha(x)]x}. \tag{4}$$

A priori, the function $\kappa(x)$ necessary to replicate I_{ion} is unknown. One can deduce that κ should be small at the leading edge of the grating (to ensure that some fraction of the light propagates to the end of the grating), and very large at the end of the grating (to minimize light leaving the end of the grating without diffracting). We can assign a smoothly varying ansatz for $\kappa(x)$:

$$\kappa(x) = ax^3 + bx^2 + cx + d + Ae^{Bx}. \tag{5}$$

We use a least-squares difference between $I_{\mathrm{diffraction}}(x)$ and $I_{\mathrm{ion}}(x)$ to determine optimal values for the coefficients $\{a, b, c, d, A, B\}$. For $\alpha(x) = 0$, the optimal coefficients identify an ideal variation of κ along the length of the grating that maximizes coupling to the ion within the etendue limit afforded by the finite length of the grating. Then we identify the realizable optimal grating strength $\kappa(x)$ by repeating the least-squares optimization subject to the constraints of non-zero excess loss α and finite diffraction strength κ_{max}. We obtain these parameters from the optimized grating parameter libraries described in the preceding section (Fig. 8a).

We combine longitudinal focusing and apodization using a lookup process. The process starts with the calculation of the required diffraction angle at a point x within the length of the grating from Snell's law or Fermat's principle of least time. Then we determine the optimal local coupling strength from our numerical fit of Eq. 5. Using the determined diffraction angle and coupling strength at a given point, we interpolate the grating pitch, optimum geometric parameters (DCU, DCL, dx), and phase-shift δ from discrete solutions in the optimized grating parameter library. To define an actual grating we discretize the continuously varying solutions at each grating tooth (see Fig. 8).

S1.4. 3D focusing

We realize focusing transverse to the axis of propagation by curving the grating lines in the xy-plane. We define the curved grating pattern using two steps. First, the previously defined 2D grating solution for longitudinal focusing

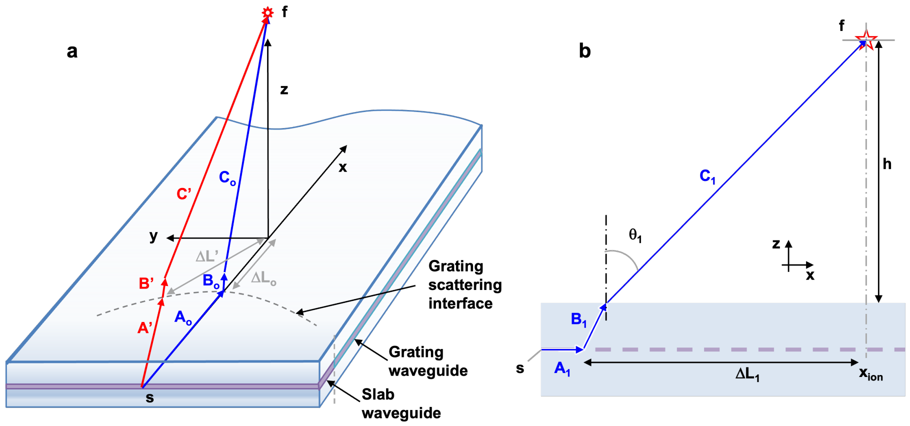

Fig. 9. Geometry for defining the curvature of grating lines for a 3D focusing grating waveguide. **a** Perspective view and **b** cross-sectional view. The curvature of each grating scattering interface in the xy plane is defined by setting the optical path length to be the same in both paths $A_oB_oC_o$ (blue) and $A'B'C'$ (red), so that a photon transiting from ‘s’ to ‘f’ on any path arrives at the same phase.

and apodization sets the location of grating interfaces on the x axis. Second, the scattering interfaces are extruded in the y direction along curves that ensure photons from a common source remain in phase at the focal point (see Fig. 9). Photons transiting the marginal path A’B’C’ (red) travel a longer physical length, but segment A’ in the slab waveguide (where the speed of propagation is slower) is shorter, such that photons transiting either ABC path arrive at ‘f’ in-phase. We derive the length of segments B and C from Snell’s law and the in-plane distance ΔL. The cumulative phase in transiting path BC is $2\pi \cdot (n_{\mathrm{SiO_2}} \cdot B + n_{\mathrm{air}} \cdot C)/\lambda_0$, with n_i the refractive index of each material i.

For the special case where the source at ’s’ has a flat phase front with a y width $\gg \lambda_m$ (an expanded collimated beam), the cumulative phase in path A is approximately invariant in y, and is simply $2\pi \cdot n_{\mathrm{slab}} \cdot (x - x_s)/\lambda_0$, where x_s is the x location of the source and n_{slab} is the effective modal propagation constant of the slab waveguide. In a more generic case, where the phase varies in y (a common case being light diverging in the plane from a narrow single-mode waveguide), the cumulative phase in path A can be determined by simulating the propagation of light in a uniform planar slab waveguide emanating from a source at ‘s’ in 3D FDTD, and then extracting and unwrapping the phase [34] of the electric field propagating in the plane. For most cases, we find that the refraction at the interface of the slab waveguide and the grating waveguide is sufficiently small such that it can be neglected in calculating the grating curvature, though this could be accounted for by recalculating the in-plane phase map in 3D FDTD after the curvature of the first grating line that defines the interface has been determined.
